# Variations in Kinematics during Clinical Gait Analysis in Stroke Patients

**DOI:** 10.1371/journal.pone.0066421

**Published:** 2013-06-17

**Authors:** Julien Boudarham, Nicolas Roche, Didier Pradon, Céline Bonnyaud, Djamel Bensmail, Raphael Zory

**Affiliations:** GRCTH, EA4497, CIC-IT 805, CHU Raymond Poincaré, Garches, France; The University of Queensland, Australia

## Abstract

In addition to changes in spatio-temporal and kinematic parameters, patients with stroke exhibit fear of falling as well as fatigability during gait. These changes could compromise interpretation of data from gait analysis. The aim of this study was to determine if the gait of hemiplegic patients changes significantly over successive gait trials. Forty two stroke patients and twenty healthy subjects performed 9 gait trials during a gait analysis session. The mean and variability of spatio-temporal and kinematic joint parameters were analyzed during 3 groups of consecutive gait trials (1–3, 4–6 and 7–9). Principal component analysis was used to reduce the number of variables from the joint kinematic waveforms and to identify the parts of the gait cycle which changed during the gait analysis session. The results showed that i) spontaneous gait velocity and the other spatio-temporal parameters significantly increased, and ii) gait variability decreased, over the last 6 gait trials compared to the first 3, for hemiplegic patients but not healthy subjects. Principal component analysis revealed changes in the sagittal waveforms of the hip, knee and ankle for hemiplegic patients after the first 3 gait trials. These results suggest that at the beginning of the gait analysis session, stroke patients exhibited phase of adaptation,characterized by a “cautious gait” but no fatigue was observed.

## Introduction

About one half of stroke survivors present with motor impairments such as: synkinesis, abnormal muscle tone and orthopaedic deformations. About 52 to 85% of hemiplegic patients regain the capacity to walk, but their gait differs from that of healthy subjects [1], [2]. Hemiplegic gait is characterized by alterations in spatio-temporal and kinematic parameters [3].

Gait analysis is frequently carried out in clinical practice i) to identify gait impairments; ii) to determine appropriate treatments, and iii) to evaluate the effectiveness of interventions [4]. Three-dimensional gait analysis is the gold standard for gait evaluation in patients with gait abnormalities [5]. It is used to simultaneously quantify spatio-temporal, kinematic, kinetic and electromyographic gait parameters. A review found moderate to good reliability for most biomechanical parameters during a gait analysis session [6]. The authors concluded that, while the level of errors is probably acceptable, they should be taken into account in the clinical interpretation of the results. It is important that data obtained with such methods identify true and significant changes in gait performance [5].

Classically, a gait analysis session in a disabled population, such as stroke patients, involves the recording of 10 gait trials at the patient’s spontaneous gait velocity. Then, the results of each gait parameter are averaged over the complete gait session. However, in a review, Lord and Rochester [7] showed that gait performance in community ambulant stroke survivors may be influenced by several factors such as lack of confidence, fear, fatigue, depression or lack of physical condition [8], [9], [10], [11]. These factors are not taken into account in the interpretation of data from a gait analysis session although they may affect the data. This hypothesis is supported by clinical observations. Indeed, clinicians frequently report that patients require time to adapt to the conditions of the gait analysis session (material used, evaluators, laboratory setting etc).

Previous studies have highlighted that fear of falling is frequent after stroke and could influence gait parameters [12], [13], [14]. In older adults, the fear of falling while walking, named “cautious gait”, leads to a specific gait pattern with reduced stride length and gait velocity along with prolonged double support time [15], [16], [17], [18], [19]. Gait variability (stride-to-stride) is also a good marker of the fear of falling [20]. Indeed, the literature clearly shows that stride-to-stride fluctuations in gait velocity, stride length and double support duration are significantly higher in patients with a “cautious gait” [15], [17], [21]. Therefore, it could be hypothesized that the adaptive phase observed by clinicians at the beginning of a gait analysis session in stroke patients could be related to “cautious gait” and therefore could influence gait parameters, particularly at the beginning of the recording session.

In addition, deconditioning and functional loss [22], [23] mainly due to inactivity may contribute to a phenomenon of fatigue [24], leading to an exacerbation of the gait impairments in individuals with stroke. Previous studies have shown that stroke patients exhibit great fatigability during gait [1], [25], [26], [27], [28]. These studies have established that, after stroke, walking performance declines over relatively short bouts of functionally-relevant ambulation [1], [25], [26], [27]. For example, they showed an alteration of mean spatio-temporal [25], [25] and kinematic parameters [27] in hemiplegic patients over a six-minute walk test. Moreover, in a study designed to induce fatigue in older people, the variability of some spatio-temporal gait parameters increased [29]. Similar results were found in patients with neurological disorders [30]. It was argued that the changes observed may be associated with both mechanisms of cardiorespiratory and muscle fatigue which influence performance [31]. These results suggest that fatigue could occur during a gait analysis session, influencing the results, particularly at the end of the recording session.

To resume, in addition to motor impairments, the environment conditions, the fear of falling, as well as fatigability, may modify the gait pattern of a population of stroke patients. These changes in gait performance, could compromise interpretation of data from gait analysis. It therefore seems relevant to evaluate the changes which occur in lower limb kinematics of hemiplegic patients across trials recorded during one gait analysis session. The results should help clinicians to focus their analysis only on gait trials which significantly reflect the patient’s gait pattern [6], [4], [32].

Therefore, the aim of this study was to determine if the gait of hemiplegic patients significantly changes over the successive gait trials of a gait analysis session (9 gait trials). To that end, both the mean and the variability of frequently evaluated spatio-temporal gait parameters and the joint kinematics of hemiplegic patients and healthy subjects were quantified, using a motion analysis system. The spontaneous mean gait velocity was chosen as the primary outcome measure to quantify changes in performance over the gait analysis session. Next, principal component analysis (PCA) was used to reduce the number of variables from the joint kinematic waveforms and to identify the regions of the gait cycle which were modified during the gait analysis session. Because of their motor impairments, we hypothesized that the gait performance of hemiplegic patients would improve after the first gait trials, following a phase of adaptation and then deteriorate as fatigue occurred. In contrast, we expected the parameters of healthy subjects to remain stable.

## Materials and Methods

### Subjects

A database of clinical gait analyses carried out between 2004 and 2012 was reviewed. Forty two chronic hemiplegic subjects were included in this study (33 males and 9 females; age: 52 (13) years; height: 172 (8) cm; mass: 72 (8) kg; time since stroke: 59 (78) months). The criteria for inclusion were: over 18 years old, hemiplegia following a stroke occurring more than 6 months prior to the gait analysis session, recordings of at least 9 gait trials carried out without the use of any assistive device, barefoot and at the patient’s comfortable velocity. In addition, 20 healthy volunteers who had no past history of neurological or musculoskeletal pathologies were recruited (9 males and 11 females; 33 (7) years; 172 (6) cm; 66 (10) kg). This study was retrospective, approved by the local Ethics committee of Ile de France XI and all subjects provided written informed consent prior to the gait analysis session, with regard to the potential use of their data in any study-type procedures.

**Figure 1:**
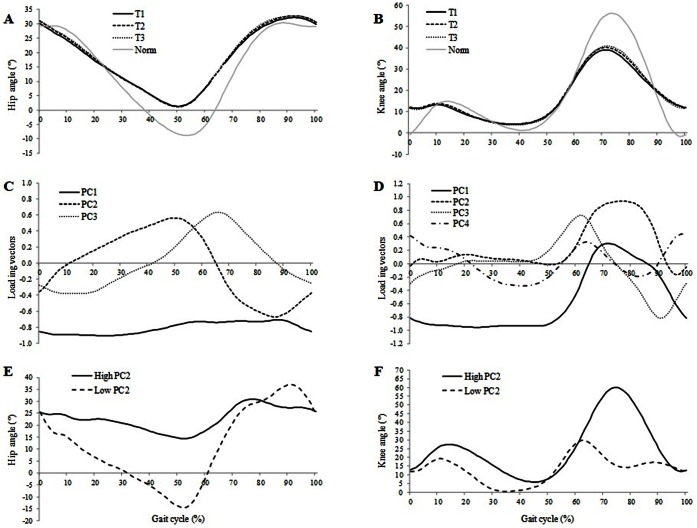
Normalized gait cycle of the mean hip (A) and knee (B) flexion/extension waveform of the paretic limb and the normal value: condition T1 (solid), condition T2 (dashed) and condition T3 (dotted). The solid grey line indicates normal values. Loading vectors for the principal components: PC1 (solid), PC2 (dashed), PC3 (dotted) and PC4 (dashed–dotted) of hip (C) and knee (D) kinematic waveforms. Higher (solid) and lower (dashed) score from PC2 for knee (E) and hip (F) flexion waveforms.

**Figure 2:**
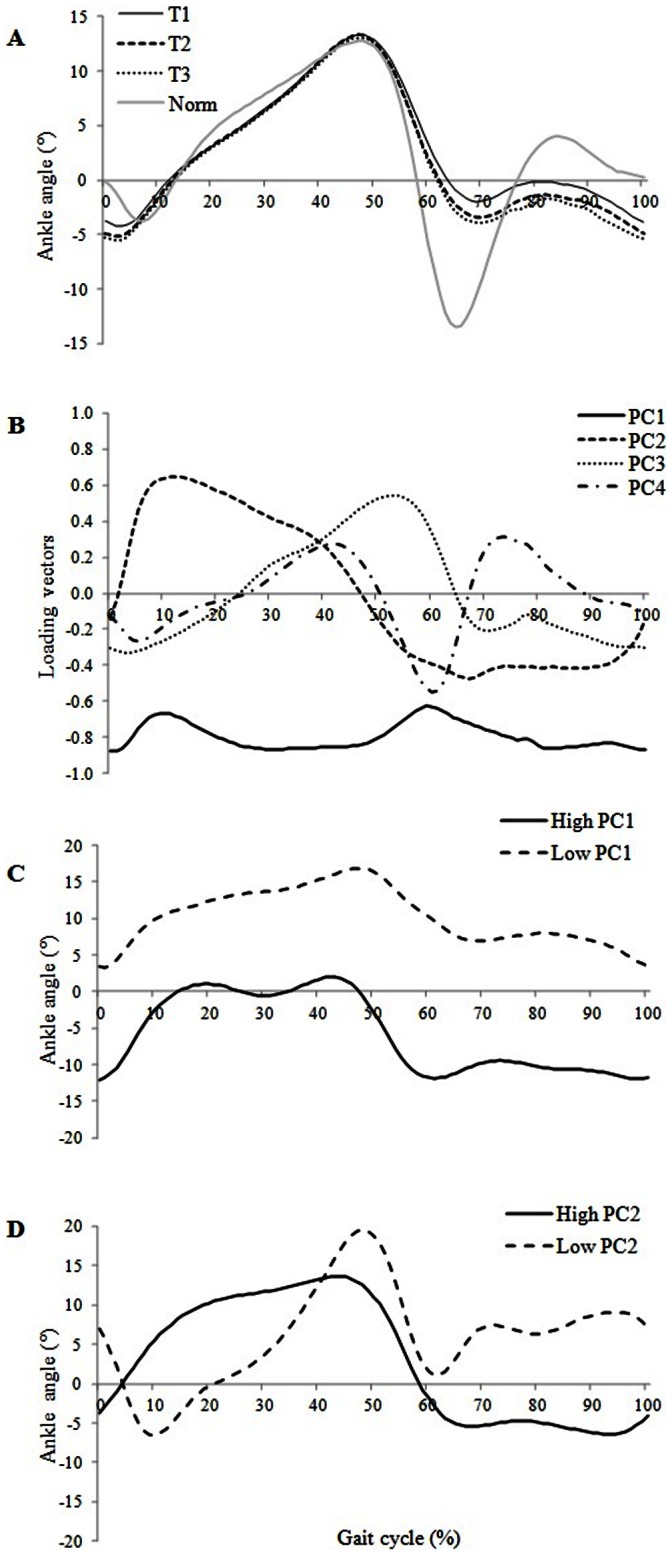
Normalized gait cycle of the mean ankle (A) flexion/extension waveform of the paretic limb and the normal value: condition T1 (solid), condition T2 (dashed) and condition T3 (dotted). The solid grey line indicates normal values. Loading vectors for the principal components, PC1 (solid), PC2 (dashed), PC3 (dotted) and PC4 (dashed–dotted) of ankle (B) kinematic waveforms. Higher (solid) and lower (dashed) score from ankle PC1 (C) and from ankle PC2 (D) flexion waveforms.

### Procedure

#### Gait analysis

Each subject (hemiplegic and healthy) carried out one gait analysis session. Each gait analysis was carried out in a 10-meter gait corridor and was composed of 9 trials with the subjects barefoot and walking at their preferred velocity. Gait was analyzed using a motion capture system with 8 optoelectronic cameras (Motion Analysis Corporation, Santa Rosa, CA, USA, sampling frequency 100 Hz). The trajectories of 30 markers placed on anatomical landmarks, using the Helen Hayes marker set [33], were collected and filtered using a fourth-order zero-lag Butterworth low-pass-filter, with a 6 Hz cutoff frequency [34]. All 9 gait trials were computed for analysis. The clinical evaluation and the preparation for the gait analysis session take about 25–30 minutes. Between each gait trial, no rest was allowed and the recording was only stopped between trials while the subjects turned around. The position of the reflective markers was not altered during the entire gait analysis session.

#### Data processing

Spatio-temporal and sagittal kinematic parameters were calculated for each gait cycle, using OrthoTrack 6.5 software (Motion Analysis Corporation, Santa Rosa, CA, USA). For each gait cycle, the spatio-temporal parameters of the paretic limb were calculated: gait velocity, stride time, stride length, cadence, step width, step length, stance phase time, swing phase time and double support time. The sagittal hip, knee and ankle kinematic waveforms for the paretic limb were time-normalized to 101 points, comprising the gait cycle from 0 to 100% with 1% increments. Only the sagittal kinematic plane was evaluated to identify the most frequent deviations described in hemiplegic gait [3], [35].

### Clinical Evaluation

Patients underwent a clinical neurological evaluation before the gait analysis. Spasticity and strength of quadriceps, hamstring and triceps surae muscles were evaluated with the Modified Ashworth Scale (MAS) and Medical Research Council (MRC) scale, respectively. These tests were performed in order to evaluate the severity of the lower limb impairment.

### Statistical Analysis

The analyses were carried out on 3 consecutive gait trials from the 9 gait trials of the gait analysis: from the first to the third gait trial (condition T1), from the fourth to the sixth gait trial (condition T2) and from the seventh to the ninth gait trial (condition T3). These three conditions were chosen following the suggestion of Maynard et al. [36] that a minimum of three gait cycles should be averaged to overcome the effects of stride-to-stride variability.

Since gait analysis results in a large quantity of kinematic data, which are often correlated, a principal component analysis (PCA) was performed with the kinematic variables of the hip, knee, and ankle in the sagittal plane. A PCA is a multivariate statistical technique that has been shown to be an effective tool in the reduction and interpretation of gait waveform data [37]. A PCA aims to reduce the information contained in the sample gait waveforms to a small number of components, named principal components (PC), which explain the majority of variance in the data through linear combinations obtained from those variables. Therefore, by transforming correlated variables into new uncorrelated variables, differences in kinematic waveforms can be detected in specific portions of the gait cycle [37]. Thus, the PCA summarizes the information contained in the gait cycle while retaining the temporal characteristics [38], [39], [40], [41], [42].

Kinematic gait data were arranged in 6 (2 groups * 3 joints) n*101 separate data matrices (n: number of gait cycles included in the analysis, and 101: time points per gait cycle). A PCA was performed on each matrix. First, an orthogonal transformation converted the 101 variables, corresponding to the 101 percentages of the gait cycle, to a limited number of PC which were uncorrelated with each other. The PC explained the majority of the variance in the original gait cycle variables. The number of PC was chosen to explain at least 90% [38] of the total data variability. A coefficient called a load vector was assigned to each variable (0 to 100%) of each PC. The load vectors weighted the importance given to each variable of each PC and represented biomechanically relevant features of the kinematic waveforms. The PC scores were computed for each of the three conditions based on the load vector, for each gait cycle of each patient, which are derived by multiplying the load vectors by the angle variables values of each individual during the gait cycle. The PC scores defined the contribution of a given PC to each subject’s kinematic waveform. In other words, for a particular patient, a set of PC scores was generated from original kinematic waveform data, representing the distance between kinematic waveform of a patient from the average of a given PC. Data interpretation of the features of a given PC was performed through load vector graphs and with the average curves of the patients who had the highest and lower scores (5^th^ and 95^th^ percentiles) [37].

Kolmogorov-Smirnov tests were conducted before the statistical analysis and confirmed that data were normally distributed. A one-factor ANOVA with repeated measures was used to analyze differences between the three conditions (T1 vs. T2 vs. T3) for each group separately (hemiplegic patients and healthy subjects) for spatio-temporal parameters and for the PC scores from the hip, the knee and the ankle kinematic waveforms. Post hoc analysis was performed using the Bonferroni post hoc multiple comparison test. The significance level was maintained at p<0.05 with Bonferroni adjustments used as appropriate. In addition, since the demographic characteristics (age, height and weight) and the clinical characteristics (spasticity, strength and time since hemiplegia) of our sample of patients were very heterogeneous, Spearman correlation tests were used to determine the strength of the relationships between these characteristics and the spatio-temporal parameters. Means, standard deviations (SD) and coefficients of variation (CV) were calculated for each parameter. Values from the two clinical evaluation scales (MAS and MRC) were expressed as medians. Statistical analysis was performed using Statistica 7 (StatSoft, Inc., Tulsa, OK, USA).

## Results

For detailed characteristics of the 42 hemiplegic patients and of the 20 healthy subjects included, see Table 1. No significant correlations were found between any of the spatio-temporal parameters and age, height, weight, degree of spasticity (MAS), strength (MRC) or time since hemiplegia.

**Table 1:** Demographic characteristics of subjects.

		Hemiplegic patients (N = 42)		Healthy subjects (N = 20)
		Description of populations		
Gender (M/F)		33/9	9/11	
Age (years)		52 (13)	33 (7)	
Height (cm)		172 (8)	172 (6)	
Weight (kg)		72 (14)	66 (10)	
Paretic side (R/L)		19/23	−	
Time since hemiplegia		59 (78)	−	
		**Clinical examination**		
**Spasticity**				
MAS	Quadriceps	1.5 [2 (0.7)]	−	
	Hamstrings	1 [1 (0.4)]	−	
	Triceps surae	2 [2 (0.8)]	−	
**Strength**				
MRC scale	Quadriceps	3 [3 (0.6)]	−	
	Hamstrings	3 [3 (0.7)]	−	
	Triceps surae	2 [3 (1.1)]	−	

Mean (SD) values of demographic characteristics of hemiplegic patients and healthy subjects. For clinical examination, median values are presented (mean and standard deviation in brackets).

M = male, F = female, R = right, L = left, MAS = Modified Ashworth Scale, MRC = Medical Research Council.

### Spatio-temporal Parameters (Table 2)

For the hemiplegic patients, mean gait velocity, stride length and cadence were significantly higher in T2 and T3 compared with T1 (p<0.01). Swing phase duration for the paretic limb was significantly shorter (p = 0.02) in T3 compared with T1. The CV of gait velocity, step length and double support time were significantly lower in T2 and T3 compared with T1 (p<0.05) and the CV of step width was significantly lower in T3 compared with T2 (p<0.01). There were no significant differences between conditions for means and CV of any of the spatio-temporal parameters of the healthy subjects.

**Table 2:** Spatio-temporal parameters for hemiplegic patients and healthy subjects.

Parameters		Spatio-temporal parameters								
		T1		T2		T3	T1		T2	T3
		Hemiplegic patients (N = 42)					Healthy subjects (N = 20)			
Gait velocity (m.s-^1^)	Mean (SD)	0.78 (0.25)	0.82 (0.26)†		0.82 (0.26)*		1.26 (0.19)	1.25 (0.18)		1.25 (0.18)
	CV (%)	6.7	5.0†		5.3*		2.8	3.0		2.8
Stride time (s)	Mean (SD)	1.35 (0.20).	1.33 (0.21)		1.32 (0.20)		1.05 (0.08)	1.06 (0.08)		1.06 (0.08)
	CV (%)	4.4	3.7		4.2		2.4	2.1		2.3
Stride length (m)	Mean (SD)	1.01 (0.20)	1.04 (0.21)†		1.03 (0.22)*		1.32 (0.13)	1.31 (0.12)		1.31 (0.11)
	CV (%)	5.3	4.6		4.7		2.7	2.6		2.5
Cadence (step.min-^1^)	Mean (SD)	91.0 (13.6)	92.7 (14.7)†		93.1 (14.5)*		114.8 (8.7)	114.2 (8.5)		114.3 (8.6)
	CV (%)	4.4	3.7		4.3		2.5	2.1		2.4
Step width (cm)	Mean (SD)	19.3 (5.4)	19.1 (5.3)		19.3 (5.3)		15.5 (2.0)	15.2 (2.3)		15.2 (2.4)
	CV (%)	8.0	10.0		6.4#		5.6	5.9		5.4
Step length (m)	Mean (SD)	0.50 (0.09)	0.52 (0.09)		0.52 (0.09)		0.65 (0.05)	0.65 (0.04)		0.65 (0.04)
	CV (%)	6.6	5.3†		5.3*		3.7	3.4		3.9
Stance phase duration (%GC)	Mean (SD)	59.7 (4.1)	59.5 (3.8)		59.8 (4.0)		60.2 (1.7)	60.4 (1.2)		60.4 (1.2)
	CV (%)	4.5	4.2		3.8		2.4	2.2		1.8
Swing phase duration (%GC)	Mean (SD)	40.3 (4.1)	40.5 (3.8)		40.2 (4.0)		39.8 (1.7)	39.6 (1.2)		39.6 (1.2)
	CV (%)	6.7	6.2		5.7		3.5	3.2		2.8
Double support duration (%GC)	Mean (SD)	28.2 (5.5)	27.5 (5.6)		27.1 (5.3)		20.3 (1.5)	20.6 (1.3)		20.6 (1.2)
	CV (%)	10.5	8.8†		8.8*		3.5	3.0		3.1

Mean spatio-temporal parameters (SD) and coefficient of variation (CV) during stance and swing phase for condition T1 (1–3 gait trials), condition T2 (4–6 gait trials) and condition T3 (7–9 gait trials). GC = gait cycle.

† Significant difference between T1 and T2 (p<0.05).

# Significant difference between T2 and T3 (p<0.05).

* Significant difference between T1 and T3 (p<0.05).

### PCA on Joint Kinematic Waveforms (Table 3, Figures 1 and 2)

#### Hip

For the hemiplegic patients, three PC were needed to explain 94% of the total variability of the hip flexion/extension angle waveforms (Table 3). The ANOVA showed that only PC2 was statistically different between T1 and T2 (p<0.01) and between T1 and T3 (p = 0.01). There were large positive values for PC2 in mid and late stance and large negative values during swing; PC2 measured range of hip motion throughout the gait cycle. High PC2 scores were associated with large differences in hip extension angle during mid and late stance (40–60% of the gait cycle) and in hip flexion angle during late swing (80–100% of the gait cycle). This means that there was an increase in hip extension angle during mid and late stance as well as an increase in hip flexion angle during late swing between T1 and T2 and T1 and T3 (p<0.01 and p = 0.01 respectively). For healthy subjects, three PC were needed to explain 96% of the total variability of the hip flexion/extension angle waveforms. The ANOVA showed no significant differences between conditions for any of the three PC.

**Table 3:** Principal component model for hemiplegic patients.

Kinematicmeasure	Variation Explained (%)	PC	Interpretation	Mean PC scores (SD)		
				T1	T2	T3
*Hip flexion angle*	66	PC1	−	0.04 (0.96)	−0.03 (1.03)	−0.01 (1.03)
	17	PC2	Range of motion of hip flexionthroughout gait cycle	0.09 (0.96)	−0.16 (0.93)†	−0.13 (1.05)*
	11	PC3	−	0.01 (1.01)	0.03 (0.96)	−0.04 (1.06)
*Knee flexion angle*	52	PC1	−	−0.02 (1.02)	−0.05 (1.02)	0.07 (0.99)
	19	PC2	Amplitude of knee flexion during swing	−0.11 (1.00)	0.16 (0.96)†	0.10 (1.01)*
	14	PC3	−	−0.08 (0.97)	0.02 (1.01)	0.06 (1.05)
	5	PC4	−	−0.02 (0.95)	0.06 (1.04)	−0.03 (1.03)
*Ankle flexion angle*	63	PC1	Magnitude of ankle flexionthroughout gait cycle	−0.11 (0.99)	0.13 (0.98)†	0.06 (1.00)*
	17	PC2	Amount of dorsiflexion in mid stanceand plantarflexion during swing	−0.14 (1.03)	0.09 (0.98)†	0.02 (1.01)*
	8	PC3	−	−0.11 (0.95)	0.07 (1.04)	0.03 (1.01)
	5	PC4	−	−0.06 (0.95)	0.08 (0.98)	−0.01 (1.07)

Principal components (PC) and mean PC score (SD) for condition T1 (1–3 gait trials), condition T2 (4–6 gait trials) and condition T3 (7–9 gait trials).

† Significant difference between T1 and T2 (p<0.05).

* Significant difference between T1 and T3 (p<0.05).

#### Knee

For the hemiplegic patients, four PC were needed to explain 90% of the total variability of the knee flexion/extension angle waveforms (Table 3). The ANOVA showed that only PC2 was statistically different between T1 and T2 (p<0.01) and between T1 and T3 (p = 0.03). PC2 had values almost equal to zero during the entire stance phase and large positive values during swing; hence, PC2 appeared to measure the amplitude of knee flexion during the swing phase of the gait cycle. Visual analysis of high and low values of PC2 scores found that a high PC2 score was associated with a large difference in knee flexion angle during mid and late swing phase (70–100% of the gait cycle). This means that that knee flexion angle during mid and late swing increased between T1 and T2 and between T1 and T3 (p<0.01 and p = 0.03 respectively). For the healthy subjects, four PC were needed to explain 90% of the total variability of the knee flexion/extension angle waveforms. The ANOVA showed no significant differences between conditions for the four PC.

#### Ankle

For the hemiplegic patients, four PC were needed to explain 93% of the total variability of the ankle flexion/extension angle waveforms. The ANOVA showed statistically significant differences for PC1 and PC2 between T1 and T2 (p<0.01) and between T1 and T3 (p = 0.02). PC1 had negative values which varied very little during the entire gait cycle; hence, PC1 seemed to measure the overall magnitude of the ankle angle throughout the entire gait cycle. PC2 had large positive values during mid stance and large negative values during swing phase; hence, PC2 appeared to be related to the amount of dorsiflexion during mid stance (10–30% of the gait cycle) and the amount of plantarflexion during swing (60–100% of the gait cycle). Visual analysis of high and low values of PC2 scores showed that high PC2 scores were associated with an increase in ankle dorsiflexion in stance particularly in mid stance and an decrease of ankle plantarflexion during swing. This means that the angle of ankle dorsi/plantarflexion during the stance and swing phases increased between T1 and T2 and between T1 and T3 (p = 0.01 and p = 0.02 respectively). For the healthy subjects, five PC were needed to explain 91% of the total variability of the ankle flexion/extension angle waveforms. The ANOVA showed no significant differences between conditions for any of the five PC.

## Discussion

The aim of this study was to determine if the gait of hemiplegic patients changes significantly over successive gait trials carried out during a gait analysis session. The major findings were that i) mean gait velocity and the other spatio-temporal parameters were significantly greater, and their variability significantly lower, during the six last gait trials compared to the first three, for hemiplegic patients but not for healthy subjects, ii) the lower gait velocity during the first three trials coincided with lower hip, knee and ankle range of motion as shown by the PCA, and iii) the gait pattern and the gait velocity were not affected by fatigue mechanisms at the end of the session.

The values of the spatio-temporal parameters for the hemiplegic patients in this study were consistent with those reported in previous studies [26], [43], [44]. Gait velocity, cadence and stride length were significantly lower during the three first trials compared to the six last trials with no differences between the middle 3 and last 3 trials. Although the increase of 4 cm/s (+5.1%) in mean gait velocity found in this study appears small, this magnitude is clinically significant. Indeed, Sibley et al. [26] showed that, in stroke patients, a 3.2 cm/s decrease in gait velocity during a six-minute walk test was clinically significant and in patients with Multiple Sclerosis, a change of 3 cm/s has been proposed to be clinically significant [45]. Moreover, the minimal detectable change (smallest amount of change in a parameter necessary to conclude that the change is not attributable to error and represents a ‘‘meaningful’’ change) for gait velocity was estimated at 5%, in a recent study in a sample of stroke patients [46], and between 4 and 6 cm/s in a sample of older people [47]. Moreover, the changes observed in the present study seem to be common to a large range of stroke patients (such as ours) since no relationship between patient characteristics and spatio-temporal parameters was found. In contrast, the gait parameters of the healthy subjects were consistent over the entire gait analysis session. This result confirms a recent study by Iosa et al. [28] who observed no change in gait velocity between the first and the last minute of a six-minute walk test in healthy subjects. This discrepancy in results could be explained by the differences in age between the healthy subjects and patients. However, even if the age difference is a potential limitation of our study, the results did not show any significant correlations between age and kinematic parameters or magnitude of changes over the gait session.

To summarize, gait velocity and other spatio-temporal parameters improved after the first three trials. This increase in mean spatio-temporal values was associated with a decrease in the variability of gait velocity, step length, step width and double support time. No changes were found for healthy subjects over the gait trials. Several hypotheses could explain this phenomenon. The first three gait trials could be described as adaptive, since the environment is new, the patient is barefoot and has a variety of materials taped on his/her lower limbs (reflective markers, and adhesive tape). Negative psychological factors such as anxiety, generally associated with fear of falling [48], may also inhibit the gait of hemiplegic patients [12] at the beginning of the evaluation. This phenomenon has been described in the literature as “cautious gait” and aims to improve balance and stability [16], [17], [18]. Indeed, Iosa et al. [28] showed that hemiplegic patients adopt a compensatory strategy based on the reduction of gait velocity in order to maintain gait stability. Studies have shown that the reduction of gait velocity is a strategy which reduces upper body accelerations [33], [49] and hence decreases the risk of falls [50]. These results support our findings of a reduction in stride-to-stride variability across gait trials for the hemiplegic patients, suggesting improved gait stability and a decreased fear of falling [51]. Moreover, Balash et al. [52] found an improvement in gait velocity and gait variability, when fear of falling was reduced in a population of older adults with severe gait impairments.

Finally, in clinical practice, many patients report difficulties in walking normally after a long rest period. The gait analyses were carried out after a period of about 25–30 minutes during which the patient carried out the clinical evaluation and was prepared for the gait analysis but did not walk. We could therefore hypothesize that this ‘rest period’ might influence gait velocity. This is, however, only supported by clinical observations since it has never been scientifically studied. These hypotheses regarding the lower gait velocity at the beginning of the gait analysis seem to be specific to the studied population, since no change was observed in the healthy subjects’ gait parameters across trials, although they were all naive to the gait analysis procedure.

Contrary to our hypothesis, the stabilization of the values of the spatio-temporal parameters (mean and CV) after the three first gait trials for the hemiplegic patients showed that fatigue did not influence the data from recorded during the gait analysis session (at least over 9 gait trials). This result is in contradiction with Sibley et al. [26] who showed that the gait velocity of hemiplegic patients decreased during the last 2 min of a six-minute walk test. This difference could result from disparities between the two protocols. First, the duration of each gait analysis session in our study was less than 4 min. Secondly, during the six-minute walk test, subjects were instructed to cover as much distance as possible in 6 minutes whereas during our gait analysis session, subjects were asked to walk at their spontaneous velocity. Despite the prevalence of fatigue and well-documented reductions in functional ambulation in the stroke community [26], a phenomenon of fatigue did not influence results of the gait analysis session, certainly because of the short duration of the session and the low intensity of the effort.

Since no changes in spatio-temporal and sagittal kinematics were observed for the healthy subjects over the gait trials, the next section will focus only on modifications in sagittal kinematic waveforms for the sample of hemiplegic patients. First, the PCA of the hip flexion/extension waveforms showed that PC2 scores were significantly higher in T1 compared with T2 and T3. This result indicates that the lower gait velocity at the beginning of the gait analysis session (T1) was associated with reduced hip extension during mid and late stance and reduced hip flexion at the end of the swing phase (PC2). Our results showed that these deficits in hip function are maximal in T1 compared to T2 and T3. This confirms previous studies showing that, in stroke patients, increased gait velocity is associated with an increase in hip extension during stance phase and in hip flexion at the end of swing phase [35], [53]. In their study, Straudi et al. [27] found that gait velocity and differences in kinematic patterns could distinguish between groups of patients during a six-minute walk test. Their results showed that the faster group of patients (0.83 m/s), with a mean gait velocity equivalent to our population at the end of the gait analysis session (T3), exhibited “normal” hip motion in stance and swing phases. Visual analysis of low and high PC2 for hip motion confirms this observation with a tendency towards a normalization of hip range of motion over the gait analysis session. Moreover, the authors demonstrated that, in addition to hip impairment, inadequate knee function was also a predictor of walking performance.

Second, another well-known kinematic disorder in hemiplegic patients is a lack of knee flexion during swing phase (stiff knee gait) [54], [55], [56]. This is a common abnormality in hemiplegia and is often related to overactivity of the rectus femoris muscle due to spasticity [57]. In our study, the PCA of the knee kinematic waveforms showed that PC2 scores were significantly lower in T1 compared with T2 and T3. This result indicates that the lower gait velocity at the beginning of the gait analysis session (T1) was associated with a reduction in knee flexion amplitude during swing phase. This result is confirmed by previous studies in stroke patients which showed that increased gait velocity is associated with an increase in knee flexion during swing phase [27], [53]. It seems that the cautious gait pattern in hemiplegic patients involves a reduced range of knee motion during swing phase.

Finally, the PCA of ankle kinematic waveforms showed that the lower gait velocity at the beginning of the gait analysis (T1) was associated with i) a smaller ankle angle throughout the gait cycle compared with T2 and T3 (PC1), ii) reduced ankle plantarflexion during swing (60–100%) and iii) reduced ankle dorsiflexion in mid stance (10–30%). These changes seem to confirm our hypothesis that during the first gait trials, patients exhibited a cautious gait. First, the reduced ankle plantarflexion during the swing phase aids toe clearance. This is an appropriate response to reduce the risk of falls, particularly since patients exhibited spasticity of the triceps surae (median of 2 on MAS) which causes equinus foot [58]. In the present study, foot equinus was exhibited by 32 patients in T1, 36 patients in T2 and 37 patients in T3 during swing phase. This increase in ankle plantarflexion during swing suggests that the ‘release’ of the ankle joint could be explained by an increase in confidence across the gait cycles. However, the increase in ankle plantarflexion, which could affect safety [12], was compensated for by concomitant increases in hip and knee joint angles during swing across the gait trials.

During the stance phase, the reduced ankle dorsiflexion (10–30%) could be due to a high level of co-activation of the ankle dorsiflexor and plantarflexor muscles. Indeed, Nagai et al. [59] showed that cautious gait is characterized by an increase in muscle co-activation at the ankle joint, reducing ankle motion during the stance phase. Hence, activity of the dorsiflexor muscles could be counteracted by abnormal activity of plantarflexors muscles (co-activation), reducing dorsiflexion. The PCA of hip, knee and ankle waveforms confirmed that during the gait analysis session, stroke patients exhibited an adaptation phase probably related to a cautious gait pattern.

### Conclusions

The results of this study clearly show that spatio-temporal parameters and sagittal kinematic waveforms change over several trials of a gait analysis session in stroke patients but not in healthy subjects. Spatio-temporal parameters, particularly gait velocity, were increased after the three first gait trials. The lower gait velocity during the first gait trials seemed to be related to a cautious gait or the fear of falling at the beginning of the gait analysis session. The decrease in gait variability over the session strengthens this hypothesis. After the adaptation phase, patients walked faster and the ranges of hip, knee and ankle motion increased then remained constant, suggesting that fatigue did not influence the gait pattern over a gait analysis session involving 9 gait trials. This study suggested that the cautious gait pattern exhibited by the hemiplegic patients during the first gait trials could compromise data interpretation. Since the aim of gait analysis for patients with gait impairments is to detect the more natural and significant gait pattern, it may be appropriate, for clinical data interpretation to be relevant, to remove the first three trials from the gait analysis, in order to exclude the adaptation phase.
